# Surgical trial design for incorporating the effects of learning: what is the current methodological guidance, and is it sufficient?

**DOI:** 10.1186/s13063-023-07265-5

**Published:** 2023-04-25

**Authors:** Neil Corrigan, Julia M. Brown, Richard Emsley, David G. Jayne, Rebecca E. A. Walwyn

**Affiliations:** 1 Leeds Institute of Clinical Trials Research, University of Leeds, Leeds, UK; 2 Department of Biostatistics and Health Informatics, Institute of Psychiatry, Psychology and Neuroscience, King’s College London, London, UK; 3 Leeds Institute of Medical Research at St James’s, University of Leeds, Leeds, UK

**Keywords:** Learning curve, Learning effects, Surgical trials, Surgical RCTs, Design

## Abstract

**Background:**

Surgical interventions are complex. Key elements of this complexity are the surgeon and their learning curve. They pose methodological challenges in the design, analysis and interpretation of surgical RCTs.

We identify, summarise, and critically examine current guidance about how to incorporate learning curves in the design and analysis of RCTs in surgery.

**Examining current guidance:**

Current guidance presumes that randomisation must be between levels of just one treatment component, and that the evaluation of comparative effectiveness will be made via the average treatment effect (ATE). It considers how learning effects affect the ATE, and suggests solutions which seek to define the target population such that the ATE is a meaningful quantity to guide practice. We argue that these are solutions to a flawed formulation of the problem, and are inadequate for policymaking in this setting.

**Reformulating the problem:**

The premise that surgical RCTs are limited to single-component comparisons, evaluated via the ATE, has skewed the methodological discussion. Forcing a multi-component intervention, such as surgery, into the framework of the conventional RCT design ignores its factorial nature.

We briefly discuss the multiphase optimisation strategy (MOST), which for a Stage 3 trial would endorse a factorial design. This would provide a wealth of information to inform nuanced policy but would likely be infeasible in this setting.

We discuss in more depth the benefits of targeting the ATE *conditional on operating surgeon experience* (CATE). The value of estimating the CATE for exploring learning effects has been previously recognised, but with discussion limited to analysis methods only. The robustness and precision of such analyses can be ensured via the trial design, and we argue that trial *designs* targeting CATE represent a clear gap in current guidance.

**Conclusion:**

Trial designs that facilitate robust, precise estimation of the CATE would allow for more nuanced policymaking, leading to patient benefit. No such designs are currently forthcoming. Further research in trial design to facilitate the estimation of the CATE is needed.

**Supplementary Information:**

The online version contains supplementary material available at 10.1186/s13063-023-07265-5.

## Introduction

The need for randomised controlled trials (RCT) in evidence-based medicine is well-established. There is a consensus in the surgical literature of accepting well-designed and well-conducted RCTs as a “gold standard” for the evaluation of comparative effectiveness of surgical interventions. However, it is also widely acknowledged that the surgical setting poses numerous practical and methodological challenges to the design and conduct of an RCT. While none of these challenges are necessarily unique to surgery, it has been argued that the specific way in which they combine may in fact be unique to the surgical setting. Perhaps due to this, surgical trials have developed their own body of methodological literature, and arguably their own distinct field of research. This is exemplified by the IDEAL guidelines [1–6].

Surgical interventions are complex [4–12]. Key elements of this complexity are the surgeon and their learning curve. They have often been at the centre of debate and discussion in the literature about how to appropriately design, analyse and interpret a surgical RCT, because of the methodological challenges that they pose.

In this paper, we focus on the learning curve. Specifically, how to appropriately incorporate the existence of the learning curve into the design and analysis of Stage/Phase 3 surgical RCTs.

In the “Background” section, we identify and summarise how learning curves and the methodological challenges that they pose to surgical trial design and analysis are characterised in the surgical trial literature. We summarise conventions for incorporating learning effects in surgical trial design and analysis. We outline how those conventions appear to have developed from a set of presumed necessary features of a surgical RCT, including randomisation with respect to only a single treatment component and the choice of estimand being the average treatment effect (ATE), which has led to an arguably misguided framing of the methodological problem to be solved.

In the section titled “Examining the current formulation of the problem and proposed solutions”, we critically examine the available literature and identify, summarise and discuss gaps in the surgical trial literature both in terms of the characterisation of the methodological problem and the proposed solutions. We argue that current methods for incorporating learning effects are attempted solutions to a misguided formulation of the problem, and are therefore inadequate.

In the section titled “Reformulating the problem”, we suggest a reformulation of the problem, and briefly discuss avenues for further research that we believe would more appropriately incorporate learning effects, improving surgical trial design and analysis.

## Background

### What are learning effects and what methodological challenges do they pose?

#### The individual’s learning curve

It is a practical inevitability that any individual surgeon must go through a period of learning before they become proficient at delivering a particular procedure, during which it is expected that “errors and adverse outcomes are more likely” [13]. The increase of an individual’s proficiency over time is commonly referred to as their “learning curve”.

A distinction is made between learning during the initial development of the procedure, where the community of innovators and early adopters learn from their initial experiences while simultaneously refining the procedure, and the learning curve of an individual surgeon who gains proficiency through the repeated performance of the fully-developed, stable procedure [9, 14–16]. The latter is what one may typically expect to encounter in a Stage/Phase 3 RCT since it is expected that the procedure has “stabilised” before such an RCT is carried out.

The following characterisation from Cook, Ramsay and Fayers [17] (pg. 255) appears to be the accepted foundation for conceptual models of the learning curve: “A learning curve...tends to be most rapid at first and then tails off over time. Three main features of a learning curve can be recognized. An initial or starting level defines where the performance level begins. The rate of learning measures how quickly a particular level of performance is reached. Last, the asymptote or expert level is the level at which performance stabilizes.”

#### The evolution of a new surgical intervention and the timing of an RCT

It appears to be unanimously accepted that a new surgical procedure initially goes through a period of development during which it undergoes “rapid”, “fluid”, “iterative” change in light of the early practical experience gained by its innovators/early adopters [6]. It is also accepted that the procedure ultimately “stabilise[s]”/“settle[s]” [9], in some sense, before wider adoption [6]. This raises the question of when the best time is to undertake a Stage/Phase 3 RCT to evaluate the effectiveness of the procedure.

An often-cited argument from Chalmers [18] is that randomised evaluation should begin with the first patient. It is argued that if clinical equipoise exists, then randomised allocation offers the patient the best chance of receiving the best treatment. However, many commentators argue that a randomised comparison undertaken during the development stage of a new intervention will quickly become obsolete as the intervention evolves [19–24]. The prevailing opinion is that the intervention should be allowed to fully develop before randomised comparisons are made. Practically this gives a lower bound on the appropriate timing for an RCT.

More practical considerations also impose an upper bound to the timing. It is widely accepted that if an RCT is attempted too late — after the “exponential adoption” of the intervention — then recruitment will be infeasible [2, 6, 21, 23, 25–27]. It is believed that the wide adoption of an intervention is congruent with a “conviction of likely efficacy” [6], which would then “upset equipoise” [23]. Similarly, if evaluation is attempted too late, then the new intervention may have already — rightly or wrongly — been discarded due to a conviction of likely lack of efficacy.

These arguments imply that there is a perceived interval during which an RCT of a new surgical intervention is appropriate: after having undergone its period of development, and before it has been adopted in wider practice. Given that an RCT will likely require the commencement of wider adoption to meet sample size requirements, it follows that many of the participating surgeons in an RCT will be unfamiliar with the experimental intervention.

#### Principal features of a surgical RCT

The perceived goal of a surgical RCT has been to emulate the approach to RCTs taken in drug evaluation. RCTs have been successfully implemented to evaluate drugs, and it is presumed that surgical interventions should be “similarly” evaluated, with many authors referring to “*the *(conventional) RCT” [19, 28–31], a parallel-group design where individual patients are randomised to interventions, most typically to either the experimental or control intervention, with the aim of estimating the average treatment effect (ATE). When evaluating complex interventions, randomisation under this design involves either randomising a single component of the complex intervention, or randomising between different whole intervention packages. We will refer to this design as *the conventional RCT*.

Given the required timing of a surgical RCT, many of the participating surgeons will be new adopters of the experimental intervention, still in the process of learning during the trial, whereas they will likely already be proficient with the standard care [2, 9, 15, 23, 27, 32, 33]. A common concern is that this disparity of expertise may distort the comparison between the interventions, “biasing” the results against the experimental intervention [2, 6, 9, 13, 15, 23, 27, 31, 33–37]. This perceived source of bias, referred to as “differential expertise bias”, is a “frequent criticism” of surgical RCTs [9], and is central to discussions about surgical RCT design.

Also, at the centre of debate and discussion about surgical trial design is the widely-acknowledged heterogeneity of surgical care [12, 38–40]. Design philosophies appear to diverge based on their stance on how to appropriately incorporate treatment heterogeneity in the conventional RCT. On the one hand, it is frequently treated as a nuisance, to be suppressed or eliminated from a trial, giving rise to suggestions such as standardisation of the interventions [19, 29, 41, 42] and restricting recruitment to a set of practitioners that are more homogeneous than the wider population [6, 7, 13, 19, 21, 23, 26, 28, 29, 31, 41–43]. On the other hand, discussions of “pragmatic” RCTs focus more on capturing the heterogeneity so that the trial is more inclusive and better reflects wider practice [2, 8, 9, 16, 20, 35].

Learning effects are one aspect of the heterogeneity of surgical care, and these competing design philosophies, coupled with the premise of emulating the conventional RCT, give rise to two clear and distinct schools of thought about how to best incorporate learning effects in surgical RCT design.

### Learning effects in current surgical trial design

A commonly suggested approach is to limit participation to only those surgeons who are no longer “learning” any of the interventions, for example by requiring that the surgeons need to have “completed”/“gone through”/“[reached] the plateau [of]” their “learning curve” [13, 21, 31], or to have “mastered” [22] the technique, to have “an acceptable level of”/“sufficient” experience [6, 28], or to have undergone “a period of training”/“run-in period” [7, 43]. Despite varying practical suggestions for how exactly to set the criteria, the common goal is the same, to ensure that “the learning occurs outside the study” [16].

An objection to this “experts-only” design is that it limits the external validity of the trial [8, 15, 16, 20, 29, 33, 36]. In wider practice not all surgeons that deliver these treatments will be expert surgeons, and so the results from such a trial may not be “mirrored by the larger surgical community” [36], and thus “aren’t useful to guide everyday surgical practice” [33]. When the goal is to evaluate effectiveness, it is argued that a more “pragmatic” approach is required [2, 3, 8, 9, 16, 20, 35].

A widely advocated alternative approach is therefore to aim to have a group of participating surgeons which is representative of those who would deliver the intervention in wider practice [9, 15, 20, 22, 33, 37]. This aligns with the aims of a “pragmatic” design, and suggests imposing no restrictions on the level of expertise required for a surgeon to participate in the trial, given that the surgeon meets any requirements to perform the intervention in general practice [9, 16, 20, 22, 37].

### Learning effects in current surgical trial analysis

Available guidance on incorporating operating surgeon experience in the analysis of a surgical RCT is given by a body of literature, stemming from Cook, Ramsay and Fayers [14, 15], which suggests that data about operating surgeons’ experience should be collected during the trial [9, 14-16, 20, 32, 33, 36]. It is recognised that the treatment effect may depend on the level of experience of the operating surgeon, and that a benefit of collecting data on operating surgeon experience is the abilty to explore this relationship by estimating the “learning effects” [14, 15, 44]. However, it is assumed that the trial is designed to target the ATE only, and that therefore analyses of the learning effects are ancilliary, not supported (and thus in particular not necessarily sufficiently powered) by the design.

Under the experts-only design, it is assumed that all surgeons in the trial have already reached the plateau of their learning curve, and that therefore the ATE will not vary with respect to operating surgeon experience within the trial population. The rationale for collecting operating surgeon experience data under this design is to check this assumption empirically, and to adjust for it if not, to address the differential expertise bias.

Under the pragmatic design, the rationale for collecting operating surgeon experience data is to demonstrate how representative the distribution of operating experience in the trial is, and to perform exploratory analyses which estimate how (if at all) the comparative effectiveness of the interventions changes with operating surgeon experience [14, 15].

## Examining the current formulation of the problem and proposed solutions

Surgical interventions comprise multiple treatment components. Typically they cannot be sufficiently described by procedure name alone. For example, two identical patients receiving the same procedure but from different surgeons, or at different centres, or with different pre- and post-operative care protocols, etc. are not receiving the same intervention, and therefore do not necessarily have the same expected outcome. The level of operating surgeon experience at the time of the operation is a prime example of one of these treatment components and is present in all surgical interventions. A patient undergoing a procedure performed by a surgeon with no previous experience with that procedure is not receiving the same intervention, and may not have the same expected outcome, as an identical patient undergoing the same procedure performed by the same surgeon once they are an expert. A surgical intervention is more accurately described in terms of both the procedure and the level of experience of the operating surgeon, e.g. “Procedure A” is more accurately described as “Procedure A performed by a surgeon who has performed Procedure A over 300 times previously”.

Current discussion about both how to characterise and address the challenges that learning curves pose is built upon the premise that the comparison to be made is with respect to only a single treatment component, typically Procedure, and that the primary target of the trial is the average treatment effect (ATE), a single value (e.g. an odds ratio, hazard ratio, or difference in means) which represents the difference in expected patient outcome if they are offered Procedure B compared to A, averaged over the target patient population.

**Fig. 1:**
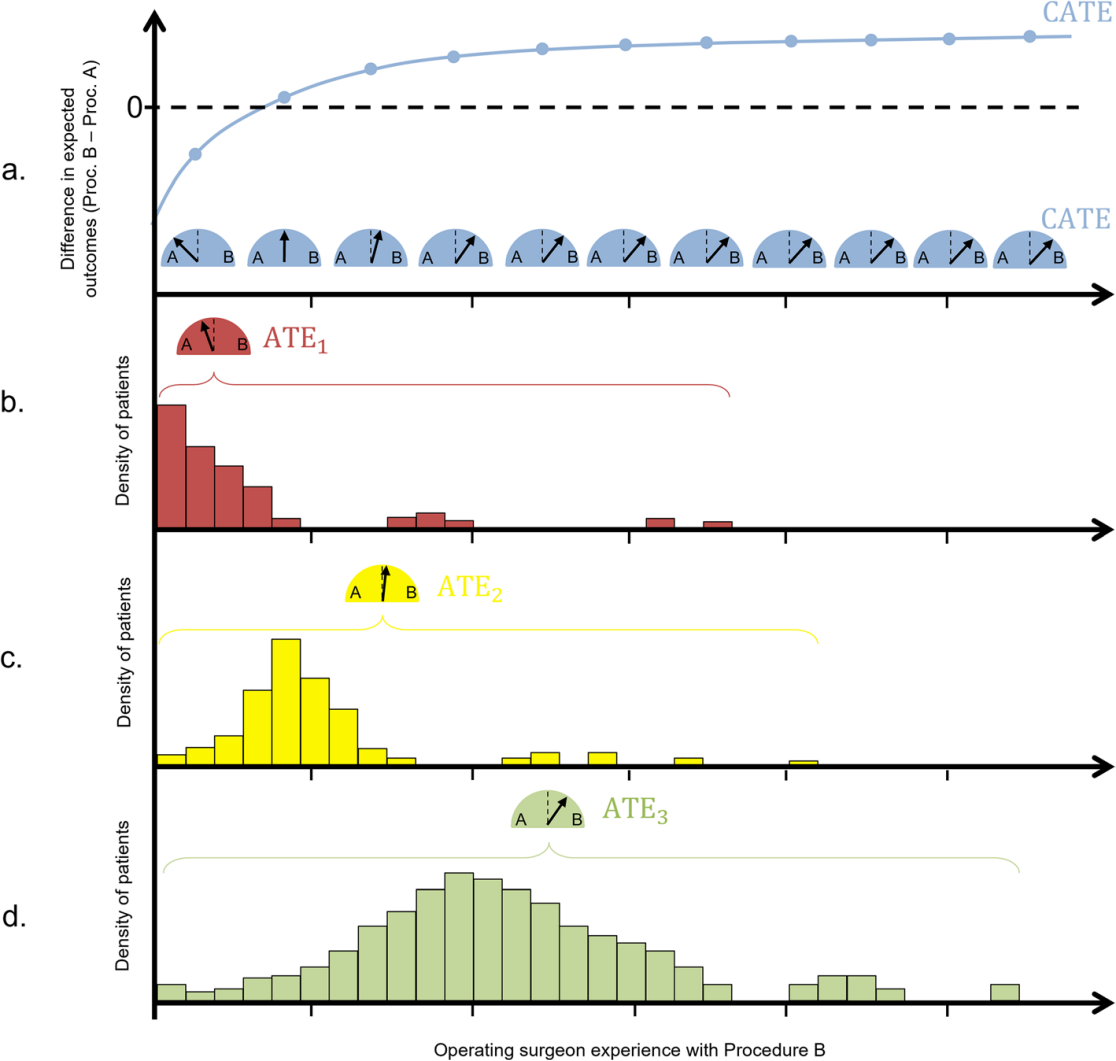
ATEs evaluating Procedure A vs B under populations with a fixed CATE but different distributions of operating surgeon experience

When making a single-component comparison only in the evaluation of multi-component interventions, the ATE represents the difference in patient outcome between the different levels of that component (e.g. Procedure A vs B) averaged over the observed levels of the other treatment components (e.g. operating surgeon and their level of experience). The hypothetical example in Fig. 1 illustrates this, where the ATE is evaluating Procedure A vs B, and does so by averaging over the observed levels of operating surgeon experience. In Fig. 1, the ATE is illustrated via the speedometer-type symbols, pointing in a single direction indicating direction and strength of superiority of a procedure. Figure 1a plots the conditional average treatment effect (labelled CATE) — the expected difference in patient outcomes under Procedure A vs B conditional on the operating surgeon’s level of experience with Procedure B. The ATE evaluated at a number of different levels of experience is illustrated in Fig. 1a. In the example illustrated in Fig. 1, Procedure B performed by a surgeon with no previous experience is inferior to Procedure A, but the comparative effectiveness of Procedure B increases under surgeons with more experience. In such a scenario, the ATE will depend on how many patients in the target population will be having their operation performed by inexperienced surgeons. That is, the ATE is specific to the distribution of the levels of experience of the operating surgeons. This is illustrated in Fig. 1b–d where the ATE is evaluated in three distinct example target populations.

The target population in Fig. 1b comprises mostly patients who would be receiving Procedure B performed by an inexperienced surgeon, and thus the ATE points to the inferiority of Procedure B. The target population in Fig. 1c comprises mostly patients who would be receiving Procedure B performed by a surgeon with moderate experience, and thus the ATE points to no difference between the Procedures. Finally, in Fig. 1d, the majority of patients would receive Procedure B from surgeons with high experience, and thus the ATE points to the superiority of Procedure B.

In summary, for a given, fixed relationship between operating surgeon experience and treatment effect, the single-component comparison of Procedure A vs B made by targetting the ATE can ultimately point to either procedure being superior, or to there being no difference between the procedures, solely depending on the distribution of the level of experience of the operating surgeon in the target population.

The methodological challenge then, as characterised by surgical trial literature, is about defining the target population such that the ATE for a randomised comparison of different levels of a single treatment component is a well-defined, consistent and meaningful quantity that can be used to guide policy in a setting where there is treatment effect heterogeneity related to operating surgeon experience. The two predominant schools of thought regarding surgical trial design represent contrasting attempts to address this challenge.

### Experts-only design

The experts-only design defines the target population as patients who are treated by surgeons who have reached the plateau of their learning curve. This approach views treatment effect heterogeneity related to operating surgeon experience as a nuisance, and attempts to eliminate it from the trial by restricting attention only to the subset of interventions where there is minimal treatment effect heterogeneity. Setting an inclusion criteria for participating surgeons in terms of a minimum threshold for their level of experience appears to be a popular approach in practice [45].

**Fig. 2:**
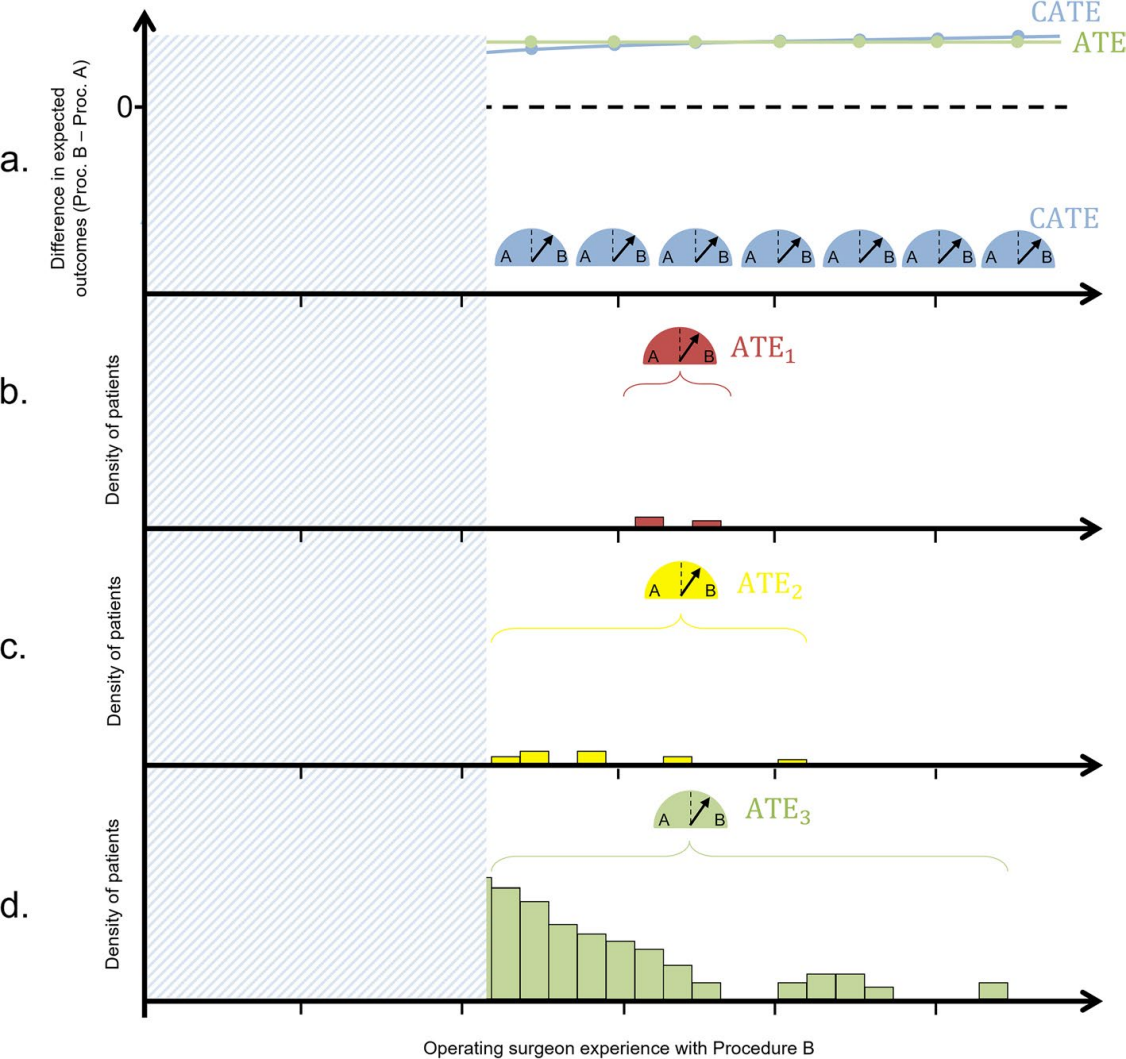
ATEs evaluating Procedure A vs B under the experts-only design in the same populations shown in Fig. 1. The shaded region represents an exclusion criteria based on (lack of) operating surgeon experience

Figure 2 illustrates the experts-only design applied to the same three example populations given in Fig. 1. Patients being treated by operating surgeons below a given threshold of experience are excluded from the trial. Consequently, in the range of experience that is included, treatment effect heterogeneity is negligible. We get the same ATE in all three target populations, but with different amounts of exclusivity of the trial population in each case.

Essentially the operating surgeon experience treatment component is being held constant at the expert level. This gives us a well-defined, consistent ATE with a valid interpretation: “How does expertly-performed Procedure B compare to expertly-performed Procedure A on average (across the population of patients who are treated by expert surgeons)?” In wider complex intervention literature, this would be referred to as the simple effect of the procedure component at the expert level of the operating surgeon experience component [46]. The results from such a trial are useful for providing evidence of efficacy — superiority of the experimental procedure under ideal conditions - but will have limited utility for evaluating effectiveness, and thus for policymaking. This is because it does not shed light on the comparative effectiveness of the procedures at any non-expert levels of operating surgeon experience.

For example, in Fig. 2, an experts-only design would reveal that Procedure B is superior to Procedure A when performed by an expert surgeon. This evidence could reasonably be used as proof-of-concept that Procedure B has the potential to be superior to Procedure A across the wider population, warranting further, more inclusive research. However, it does not act as sufficient evidence of the effectiveness of Procedure B in wider practice. For example, in the target population in Fig. 2a, most patients in practice would receive Procedure B from a relatively inexperienced surgeon, under which Procedure A is the superior choice. At best, the experts-only design in that scenario has provided unbiased information to be used for policymaking for a small fraction of the patient population, leaving the question of comparative effectiveness and safety under non-expert surgeons unanswered, but warranting further research into the effectiveness of Procedure B. At worst, the results will be used naively to inform a policy which implements Procedure B in favour of Procedure A at all levels of operating surgeon experience in practice, potentially to the detriment of the majority of patients.

### Pragmatic design

The pragmatic approach aims to sample a distribution of operating surgeon experience that is representative of wider practice, allowing, rather than suppressing, variation in the surgeons’ expertise. The rationale for this is clear: to ensure that the trial mirrors, and thus produces results applicable to, wider surgical practice. The pragmatic design aims to evaluate the effect of procedure averaged over a representative mix of the operating surgeon experience treatment component.

This yields an ATE with a seemingly useful interpretation for policymaking, i.e. “How does Procedure B compare to Procedure A on average across the whole population of patients who will be receiving the interventions in practice?” In wider complex intervention literature, this would be referred to as the main effect of the procedure component [46]. Despite this being more inclusive than experts-only design, it still has major limitations for policymaking.

Firstly, the ATE provides a single answer, pointing to only one level of the component being randomised and promoting a one-size-fits-all policy. In the 3 example target populations in Fig. 1, a “pragmatic” trial would arrive at an estimated ATE that would point to a policy of either “always Procedure A” or “always Procedure B” which, in all 3 example target populations, is not the best procedure for all patients.

Furthermore, the distribution of operating surgeon experience in the target population is not static. With every procedure that is performed, surgeons are gaining more experience, and the distribution of experience across the population is changing. For example, given the necessary timing of a trial of a new surgical intervention, one may expect that during the trial the distribution of operating surgeon experience with the experimental intervention across the wider surgeon population may look like that in Fig. 1a, with most surgeons inexperienced. By the end of the trial they will all have gained experience, shifting the original distribution (Fig. 1b), and so by the time the intervention would be rolled out in practice, the distribution is already different to what was observed in the trial. Finally, if the intervention is rolled out after the trial and becomes the new standard, then that distribution is likely to change again (e.g. Fig. 1c), and continue to change over time as new surgeons pick up the procedure and others continue to gain experience all at different rates to one another. A “representative sample” of participating surgeons during an RCT is only a snapshot of a moving target with respect to operating surgeon experience.

The strategy of the pragmatic trial to capture a representative sample of the population at the time of the trial is no guarantee that the results will be applicable to wider practice. It gives a one-size-fits-all answer that can be misleading, and nevertheless is vulnerable to obsolescence as the level of experience of operating surgeons changes over time.

### The conventional RCT design is not fit-for-purpose

The fundamental issue with the current approaches to surgical trial design is that, in a setting where the comparative effectiveness of procedure depends on operating surgeon experience, they are solutions to a flawed formulation of the problem. That is, they are attempts to make the results from the conventional RCT — a single-component comparison, evaluated via the ATE — a useful quantity for policymaking in a setting where it is arguably not fit-for-purpose.

Restricting the comparison to different choices of a single component such as procedure limits the possibilities for estimation to either a simple effect (such as the experts-only design) or a main effect (such as the pragmatic design).

In summary, in the presence of treatment effect heterogeneity associated with the level of operating surgeon experience, the ATE is dependent on the distribution of operating surgeon experience, which changes over time, and is liable to change substantially from what is observed in a trial. Nevertheless, the ATE provides a single answer, promoting a one-size-fits-all policy which, in a setting where the best policy depends on the level of experience of the operating surgeon, may lead to less-pronounced or negligible benefit, or potentially harm, in a subpopulation of patients. Unless it can be reasonably assumed that the comparative effectiveness of Procedure does not depend on operating surgeon experience in any clinically meaningful way (for example, the interventions being researched are all technically very easy, with demonstrably short/shallow learning curves), the evaluation offered by the conventional RCT lacks the nuance required for policymaking for a surgical intervention.

## Reformulating the problem

The premise that surgical RCTs are limited to single-component comparisons, evaluated via the ATE, has skewed the methodological discussion. Forcing a multi-component intervention such as surgery into the framework of the conventional RCT design ignores its factorial nature. This limits the research questions that can be addressed and therefore the usefulness of the trial for policymaking. When there is plausible treatment effect heterogeneity we should target estimands that reveal more than just the simple or main effects of the procedure, which can be used for more nuanced policymaking, ultimately to the patients’ benefit.

It would be ideal to be able to target the causal effects of both procedure and operating surgeon experience, and of their interaction. That is, for a given patient, what are the individual and combined effects of procedure and operating surgeon experience on expected outcomes. Unbiased, robust estimates of these estimands would open up a huge range of possibilities for policymaking. As well as answering both the experts-only and pragmatic trial questions, it would also allow us to answer “what is the effect of sending the patient to a more (or less) experienced surgeon?” and also “what is the best combination of procedure and operating surgeon experience to give to a patient?” With this information, policies could be tailored to any specific operating surgeon experience.

Estimating causal effects of individual components of a complex intervention when they can all be randomised is a well-established goal in experimental design, with well-developed trial design methodology. The multiphase optimisation strategy (MOST) for behavioural interventions exemplifies this [47], and outlines trial design methodology that could be directly applied to surgical interventions to answer questions about the causal effects of operating surgeon experience. However, to apply the factorial designs outlined in MOST in this setting would require us to be able to randomly allocate both the procedure and the level of operating surgeon experience. This in particular requires us to be able to directly control and stipulate the level of operating surgeon experience for any individual patient. This may indeed be possible in some circumstances and, if that is the case, then the MOST framework would be a great improvement on current approaches to surgical trial designs.

However, in practice, it is likely that we could not randomly allocate a level of operating surgeon experience to a patient, but would be entirely unable to control the level of operating surgeon experience for any individual patient’s operation. There may be constraints on which surgeon(s) an individual patient could be treated by, and constraints on the timing of the patient’s operation. A more likely scenario is one where the level of operating surgeon experience for any individual patient’s operation must be treated as a given rather than as a directly manipulable component in the design.

The current approaches to design advocated in the surgical trial literature and the fully-randomised factorial approach advocated by MOST represent two extremes. The former is entirely feasible but limited while the latter gives a wealth of information but is likely infeasible in this setting. There is however an intermediate approach to design that should be considered which would still offer an improvement over current designs while also being practically realistic.

### Trial design to target the conditional average treatment effect (CATE)

We now consider the c*onditional average treatment effect* (CATE) [48, 49], a relevant estimator for exploring treatment effect heterogeneity also referred to as moderation, statistical interaction and differential effect [50]. For a given measure of operating surgeon experience, *X* say, the CATE represents the difference in expected patient outcome if they are offered Procedure B compared to if they are offered Procedure A, averaged over the population of patients who are treated by surgeons with experience $X=x$. In a single analysis, the CATE can be evaluated for a range of values of *x* via estimation of the interaction between X and the ATE, and so rather than reducing the comparison of interventions to a single value — the ATE — we could instead have an estimate of how the procedures compare given different levels of operating surgeon experience, potentially giving different answers at different levels of operating surgeon experience.

Regarding policymaking, the CATE first asks “how experienced is the surgeon going to be for this patient’s operation?” and then points to which procedure is the better option given the answer. Note that the CATE not only asks a question about the surgeon, but also implicitly asks questions about the patient, since the population of patients who would be treated by an inexperienced surgeon likely does not have the same characteristics as the population of patients who would be treated by the most experienced surgeons [51]. In particular this means that unlike the effects estimated under a fully-randomised design, the CATE cannot be used to make causal inferences about what would happen if we sent an individual patient to a more (or less) experienced surgeon. What can be done with the CATE is weaker than what can be done with the causal effects of operating surgeon experience, but if found to be credible, could be more informative than the ATE. The CATE, unlike the ATE, allows us to recognise that the best procedure depends on the context in which the procedure will be taking place, specifically on how experienced the surgeon will be and thus also, implicitly, on what kind of patient we are treating in terms of operative difficulty.

For example, in Fig. 1, if a trial was designed to target the CATE, then it would produce results that shed light on the fact that Procedure B is relatively harmful in the population of patients being treated by surgeons with low levels of experience, is no different to Procedure A in the population of patients being treated by surgeons with moderate levels of experience, and is superior in the population of patients being treated by surgeons with high levels experience. This could lead to a policy where Procedure B is adopted by highly experienced surgeons (and/or high-volume/specialist centres) only, or a policy where Procedure B is adopted for everyone but with additional measures put in place to mitigate the evident relative harm that is caused by Procedure B under less experienced surgeons, such as supervision by an expert surgeon or a more stringent selection of which cases are given to surgeons who are still learning.

Furthermore, unlike the ATE, the CATE does not depend on the specific distribution of operating surgeon experience in the target population. For example in Fig. 1, the curve labelled CATE, which describes how the treatment effect changes conditional on operating surgeon experience, is the same for all three of the example target populations in that figure. An estimate of the CATE remains consistent as the distribution of operating surgeon experience changes. In particular, an estimate of the CATE would remain valid if the distribution of operating surgeon experience changed substantially, and so it does not share the vulnerability of the ATE to become obsolete over time due to changing distribution of operating surgeon experience.

Even in circumstances where the treatment effect may not depend on operating surgeon experience, an analysis targetting the CATE could confirm this empirically, rather than relying on an assumption, as would be the case using the ATE.

The value of estimating the CATE is recognised in the current literature [14, 15, 44], but discussion is limited to analysis methods only, while trial design unanimously targets the ATE. Under designs which target the ATE, there are no measures in place to ensure that that the analysis of the CATE will be robust and have sufficient precision to make meaningful conclusions. Consequently, estimation of the CATE is typically an auxilliary (exploratory/sensitivity) analysis only, if it is performed at all, and unlikely to provide definitive evidence to guide policymaking.

The precision of the CATE can be affected by numerous features of the design [47, 52], including the total number of patients randomised, the number of participating surgeons, the within- and between-surgeon spread of observed operating surgeon experience during the trial, the (im)balance in the numbers of patients treated by each surgeon, and the (im)balance between the treatment arms both within each surgeon and within levels of operating surgeon experience. These features can all be controlled through the design, even if the level of operating surgeon experience for any given individual patient cannot be. Crucially, the precision of the CATE estimator could be increased while holding the total sample size constant, via manipulation of the other design features.

For example, purposeful recruitment of participating surgeons, such as prioritising surgeon recruitment to target surgeons with specific levels of experience at certain times during the trial as needed, could be used to manipulate the within- and between-surgeon spread of experience observed in the trial, and therefore affect the precision of the CATE estimator. Similarly, stratification of the randomisation with respect to a measure of surgeon experience could address potential imbalances of treatment allocation in any given region of experience, again affecting the precision of the CATE estimator.

Furthermore, there are a number of established features of the trial design, sampling method and contextual considerations which can affect the credibility of the CATE estimator [50, 53]. These include considerations such as using a continuous measure for the non-randomised component rather than categorical, stratifying the randomisation by the non-randomised component, and stating an a priori hypothesis regarding the nature of the CATE (e.g. the direction in which the ATE changes with respect to the non-randomised component).

Incorporating operating surgeon experience in the primary analysis of comparative effectiveness by estimating the CATE should be welcomed. It is in the trial design where the foundation for these analyses must be laid, with a treatment allocation procedure, sampling strategy and sample size target that mitigate the biases and imprecision that the analysis would otherwise be vulnerable to.

Trial designs which facilitate the estimation of the CATE represent a clear gap in current surgical trials methodology. Specifically, there is a gap in surgical trials guidance both in terms of the recognition of the need for such designs and also regarding practically how to maximise precision of the CATE. We believe that with further research there are opportunities to design surgical trials which focus on the CATE without requiring prohibitive sample sizes, via manipulation of features other than the total sample size which affect the precision of the CATE.

## Conclusion

We have identified and summarised the current framing of the methodological problem posed by learning effects to trial design and analysis for Stage/Phase 3 surgical RCTs. We have critically examined this framing and the solutions to it advocated in the current literature. We have argued that the ATE yielded by the conventional RCT simply cannot provide adequate information for policymaking in the presence of treatment effect heterogeneity with repsect to operating surgeon experience.

We have discussed characterising the intervention explicitly as the combination of treatment components, e.g.“procedure $\times$ operating surgeon experience”, and have pointed to the theoretically ideal solution of fully-randomising the intervention to estimate causal effects of each individual component as well as their interaction.

Finally, we have briefly discussed an avenue of further research. Trial design approaches that facilitate robust, precise estimation of the conditional average treatment effect (CATE) would allow for more nuanced policymaking that will ultimately benefit patients. No such designs are currently forthcoming. Further research of what can practically be done in the trial design to facilitate the estimation of the CATE is needed.

## Supplementary information

**Additional file 1.**

## Data Availability

Not applicable.

## Abbreviations

ATE: Average treatment effect
CATE: Conditional average treatment effect
RCT: Randomised controlled trial
MOST: Multiphase optimisation strategy
