# Supplementary material for: Surgical trial design for incorporating the effects of learning: what is the current methodological guidance, and is it sufficient?
Source: Trials. 2023 Apr 25;24:294. doi: 10.1186/s13063-023-07265-5 (PMC10127059; doi:10.1186/s13063-023-07265-5)
Supplement: Supplementary file 1 — Additional file 1. [file 13063_2023_7265_MOESM1_ESM.docx]

**Literature review methods**

The literature search aimed to capture key papers from either the surgical or trials methodology literature which discuss the perceived problems that learning effects create in relation to RCT design and analysis, and/or discuss any suggested approaches for mitigating those problems. The aim was not to capture all instances of an RCT which analysed learning effects, but rather to capture high-level commentaries/discussions by surgeons and trial methodologists about the challenges of surgical RCTs, specifically relating to learning effects.

Papers were considered to be eligible for review if they included discussion of surgical learning effects in the context of RCTs. For example, commentaries about the implications of learning effects for RCT design and analysis, critiques of RCTs in surgery due to the problems that learning effects create, RCT designs to address learning curves, suggested approaches for analysing learning effects within an RCT. Papers that were the protocol, analysis plan or results from a specific RCT were excluded.

Citation searching (aka snowballing) was used to screen for eligible papers. Both backwards searching, where all of the references of a known eligible paper are screened for eligibility, and forwards searching, where all papers citing a known eligible paper are screened or eligibility, were used. Web of Science was used to compile lists of citations/citing papers from any given paper, which were exported to EndNote. In EndNote, the titles and abstracts were used as an initial screening for ineligibility. Those papers not excluded based on their title and abstract were then read and assessed for eligibility based on their content.

The starting point for these searches was Ergina et al’s 2009 Lancet paper, Challenges in evaluating surgical innovation ([1](#_ENREF_1)). This was the second in the 2009 “IDEAL” series of papers produced by the Baliol collaboration ([1-3](#_ENREF_1)). This series of papers was the product of discussions between clinicians and methodologists at conferences at the Baliol College, University of Oxford, between 2007-2009 on the topic of surgical innovation and evaluation. The Ergina paper outlines the perceived challenges to the study design of RCTs in the surgical setting. It discusses the nature of surgical procedures and how surgeon-related factors, in particular the surgeon’s level of experience and expertise, can complicate the evaluation of a surgical intervention. It was considered to be an exemplar of the eligibility criteria as described above and, due to its high-level discussion of RCTs in surgery, it would naturally cite, and be cited by, a broad range of other relevant papers.


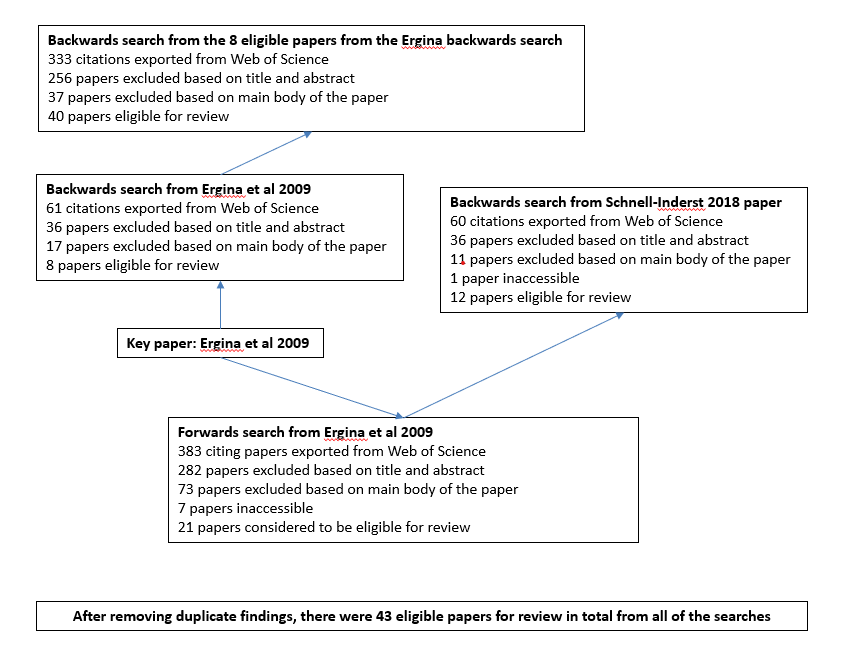


A backwards search from the Ergina paper was performed, with 8 eligible papers found within the 61 citations exported from Web of Science. A further backwards search from each of those 8 eligible papers was then performed, with 40 eligible papers found within the 333 citations exported from Web of Science. A forwards search from the Ergina paper was also performed, finding 21 eligible papers within 383 citing papers exported from Web of Science. The Schnell-Inderst 2018 paper ([4](#_ENREF_4)) was amongst these 21 eligible papers from the forwards search. It provides a detailed discussion about the challenges of surgical RCT design and analysis, and cites opinions and ideas from several papers that were not captured in the searches already performed. Given this, I performed a backwards search from the Schnell-Inderst paper, to identify this separate body of literature. This found 12 eligible papers (9 of which had already been captured in the previous searches) within the 60 citations exported from Web of Science. Pooling all of the eligible papers found, and removing duplicates, the searches identified a total of 43 eligible papers for review.

Papers included in review were published between 1980 and 2022.

A full list of papers included in the literature review is given below ([1-47](#_ENREF_1)).

1. Ergina PL, Cook JA, Blazeby JM, Boutron I, Clavien P-A, Reeves BC, et al. Surgical Innovation and Evaluation 2 Challenges in evaluating surgical innovation. Lancet. 2009;374(9695):1097-104.

2. Barkun JS, Aronson JK, Feldman LS, Maddern GJ, Strasberg SM, Balliol C. Surgical Innovation and Evaluation 1 Evaluation and stages of surgical innovations. Lancet. 2009;374(9695):1089-96.

3. McCulloch P, Altman DG, Campbell WB, Flum DR, Glasziou P, Marshall JC, et al. Surgical Innovation and Evaluation 3 No surgical innovation without evaluation: the IDEAL recommendations. Lancet. 2009;374(9695):1105-12.

4. Schnell-Inderst P, Hunger T, Conrads-Frank A, Arvandi M, Siebert U. Recommendations for primary studies evaluating therapeutic medical devices were identified and systematically reported through reviewing existing guidance. Journal of Clinical Epidemiology. 2018;94:46-58.

5. Obermair A, Simunovic M, Janda M. The impact of team familiarity on surgical outcomes in gynaecological surgery. Journal of Obstetrics and Gynaecology.

6. Vanderlinden W. PITFALLS IN RANDOMIZED SURGICAL TRIALS. Surgery. 1980;87(3):258-62.

7. Stirrat GM, Farndon J, Farrow SC, Dwyer N. THE CHALLENGE OF EVALUATING SURGICAL-PROCEDURES. Annals of the Royal College of Surgeons of England. 1992;74(2):80-4.

8. Pollock AV. SURGICAL EVALUATION AT THE CROSSROADS. British Journal of Surgery. 1993;80(8):964-6.

9. Haines A, Iliffe S. INNOVATIONS IN SERVICES AND THE APPLIANCE OF SCIENCE. British Medical Journal. 1995;310(6983):815-6.

10. Russell I. EVALUATING NEW SURGICAL-PROCEDURES - NEEDS COLLABORATION BETWEEN SURGEONS AND TRIALISTS. British Medical Journal. 1995;311(7015):1243-4.

11. McLeod RS, Wright JG, Solomon MJ, Hu XH, Walters BC, Lossing A. Randomized controlled trials in surgery: Issues and problems. Surgery. 1996;119(5):483-6.

12. Bonchek LI. Randomised trials of new procedures: problems and pitfalls. Heart. 1997;78(6):535-6.

13. Howes N, Chagla L, Thorpe M, McCulloch P. Surgical practice is evidence based. British Journal of Surgery. 1997;84(9):1220-3.

14. Mowatt G, Bower DJ, Brebner JA, Cairns JA, Grant AM, McKee L. When is the 'right' time to initiate an assessment of a health technology. International Journal of Technology Assessment in Health Care. 1998;14(2):372-86.

15. McLeod RS. Issues in surgical randomized controlled trials. World Journal of Surgery. 1999;23(12):1210-4.

16. Sauerland S, Lefering R, Neugebauer EAM. The pros and cons of evidence-based surgery. Langenbecks Archives of Surgery. 1999;384(5):423-31.

17. Campbell M, Fitzpatrick R, Haines A, Kinmonth AL, Sandercock P, Spiegelhalter D, et al. Framework for design and evaluation of complex interventions to improve health. British Medical Journal. 2000;321(7262):694-6.

18. Ramsay CR, Grant AM, Wallace SA, Garthwaite PH, Monk AF, Russell IT. Statistical assessment of the learning curves of health technologies. Health technology assessment (Winchester, England). 2001;5(12):1-79.

19. McCulloch P, Taylor I, Sasako M, Lovett B, Griffin D. Randomised trials in surgery: problems and possible solutions. British Medical Journal. 2002;324(7351):1448-51.

20. Meakins JL. Innovation in surgery: the rules of evidence. American Journal of Surgery. 2002;183(4):399-405.

21. Wente MN, Seiler CM, Uhl W, Buchler MW. Perspectives of evidence-based surgery. Digestive Surgery. 2003;20(4):263-9.

22. Cook JA, Ramsay CR, Fayers P. Statistical evaluation of learning curve effects in surgical trials. Clinical trials (London, England). 2004;1(5):421-7.

23. Lilford R, Braunholtz D, Harris H, Gill T. Trials in surgery. British Journal of Surgery. 2004;91(1):6-16.

24. Stirrat GM. Ethics and evidence based surgery. Journal of Medical Ethics. 2004;30(2):160-5.

25. Devereaux PJ, Bhandari M, Clarke M, Montori VM, Cook DJ, Yusuf S, et al. Need for expertise based randomised controlled trials. British Medical Journal. 2005;330(7482):88-91.

26. Cook JA, Ramsay CR, Fayers P. Using the literature to quantify the learning curve: A case study. International Journal of Technology Assessment in Health Care. 2007;23(2):255-60.

27. Schroeder TV. Evidence-based medicine in rapidly changing technologies. Scandinavian Journal of Surgery. 2008;97(2):100-4.

28. Cook JA. The challenges faced in the design, conduct and analysis of surgical randomised controlled trials. Trials. 2009;10.

29. Neugebauer EAM, Becker M, Buess GF, Cuschieri A, Dauben HP, Fingerhut A, et al. EAES recommendations on methodology of innovation management in endoscopic surgery. Surgical Endoscopy and Other Interventional Techniques. 2010;24(7):1594-615.

30. Sedrakyan A, Marinac-Dabic D, Normand S-LT, Mushlin A, Gross T. A Framework for Evidence Evaluation and Methodological Issues in Implantable Device Studies. Medical Care. 2010;48(6):S121-S8.

31. Bergqvist D, Rosen M. HEALTH TECHNOLOGY ASSESSMENT IN SURGERY. Scandinavian Journal of Surgery. 2012;101(2):132-7.

32. Diener MK, Simon T, Buchler MW, Seiler CM. Surgical evaluation and knowledge transfer-methods of clinical research in surgery. Langenbecks Archives of Surgery. 2012;397(8):1193-9.

33. Cook JA, McCulloch P, Blazeby JM, Beard DJ, Marinac-Dabic D, Sedrakyan A, et al. IDEAL framework for surgical innovation 3: randomised controlled trials in the assessment stage and evaluations in the long term study stage. Bmj-British Medical Journal. 2013;346.

34. Ergina PL, Barkun JS, McCulloch P, Cook JA, Altman DG, Grp I. IDEAL framework for surgical innovation 2: observational studies in the exploration and assessment stages. Bmj-British Medical Journal. 2013;346.

35. McCulloch P, Cook JA, Altman DG, Heneghan C, Diener MK, Grp I. IDEAL framework for surgical innovation 1: the idea and development stages. Bmj-British Medical Journal. 2013;346.

36. Bernard A, Vaneau M, Fournel I, Galmiche H, Nony P, Dubernard JM. Methodological choices for the clinical development of medical devices. Medical Devices-Evidence and Research. 2014;7:325-34.

37. Ceelen WP. Clinical Research in Surgery: Threats and Opportunities. European Surgical Research. 2014;53(1-4):95-107.

38. Blencowe NS, Brown JM, Cook JA, Metcalfe C, Morton DG, Nicholl J, et al. Interventions in randomised controlled trials in surgery: issues to consider during trial design. Trials. 2015;16.

39. Papachristofi O, Jenkins D, Sharples LD. Assessment of learning curves in complex surgical interventions: a consecutive case-series study. Trials. 2016;17.

40. Conroy EJ, Rosala-Hallas A, Blazeby JM, Burnside G, Cook JA, Gamble C. Randomized trials involving surgery did not routinely report considerations of learning and clustering effects. Journal of Clinical Epidemiology. 2019;107:27-35.

41. Conroy EJ, Rosala-Hallas A, Blazeby JM, Burnside G, Cook JA, Gamble C. Funders improved the management of learning and clustering effects through design and analysis of randomized trials involving surgery. Journal of Clinical Epidemiology. 2019;113:28-35.

42. Hirst A, Philippou Y, Blazeby J, Campbell B, Campbell M, Feinberg J, et al. No Surgical Innovation Without Evaluation Evolution and Further Development of the IDEAL Framework and Recommendations. Annals of Surgery. 2019;269(2):211-20.

43. Oberkofler CE, Hamming JF, Staiger RD, Brosi P, Biondo S, Farges O, et al. Procedural Surgical RCTs in Daily Practice Do Surgeons Adopt Or Is It Just a Waste of Time? Annals of Surgery. 2019;270(5):727-34.

44. Roberts DJ, Zygun DA, Ball CG, Kirkpatrick AW, Faris PD, James MT, et al. Challenges and potential solutions to the evaluation, monitoring, and regulation of surgical innovations. Bmc Surgery. 2019;19(1).

45. Siy AB, Rendell VR, Winslow ER. Analysis of National Presentations of Surgical Case Series Discussions: What Matters to Surgeons? Journal of Surgical Research. 2019;238:240-7.

46. Alsagheir A, Koziarz A, Belley-Cote EP, Whitlock RP. Expertise-based design in surgical trials: a narrative review. Canadian Journal of Surgery. 2021;64(6):E594-E602.

47. Conroy EJ, Blazeby JM, Burnside G, Cook JA, Gamble C. Managing clustering effects and learning effects in the design and analysis of randomised surgical trials: a review of existing guidance. Trials. 2022;23(1).
